# The effect of intraoperative hypotension on postoperative delirium: a meta-analysis and systematic review

**DOI:** 10.3389/fmed.2025.1690490

**Published:** 2026-01-06

**Authors:** Xiaowei Yin, Huolin Zeng, Qian Li, Qian Li, Hui Yang, Jin Liu

**Affiliations:** Department of Anesthesiology, West China Hospital, Sichuan University, Chengdu, China

**Keywords:** intraoperative hypotension, IOH, meta-analysis, POD, postoperative delirium

## Abstract

**Background:**

Postoperative delirium (POD) is a common complication linked to poor outcomes, yet its relationship with intraoperative hypotension (IOH) remains unclear. The objective of this study was to clarify the association between intraoperative IOH and POD.

**Method:**

We searched four databases (PubMed, EMBASE, Cochrane Library, and Web of Science) from their inception to June 14, 2025. Randomized controlled trials (RCTs) and observational studies were included when IOH was incorporated as a predictive variable for POD in adult patients undergoing elective surgery under general anesthesia. Risk ratio (RR) and odds ratio (OR) were calculated using a random-effect model separately in RCTs and observational studies.

**Results:**

In total, thirty out of 1,261 studies were included for the systematic review, of which 18 studies were eligible for quantitative meta-analysis. The remaining 12 studies were excluded due to incompatible data formats: eight used continuous metrics, and four lacked extractable effect estimates. IOH was significantly associated with an elevated risk of POD in both RCTs (RR: 1.89, 95%CI: 1.31–2.74) and observational studies (OR: 2.48, 95%CI: 1.64–3.75). Subgroup analysis of observational studies revealed that IOH defined by absolute threshold (OR: 4.11, 95%CI: 2.05–8.24) and mean arterial pressure (MAP) (OR: 2.90, 95%CI: 1.56–5.39) was related to a higher risk of POD. This heterogeneity was further explored by meta-regression, which identified the threshold nature of the IOH definition as a significant source of heterogeneity and a key effect modifier (*p* = 0.048).

**Conclusion:**

Our meta-analysis demonstrates a statistically significant association between IOH and increased risk of POD. However, substantial methodological heterogeneity across the included studies limits the robustness of these findings. The current evidence should therefore be interpreted as exploratory, highlighting the need for more standardized investigations in this field.

**Systematic review registration:**

Identifier CRD42023424166.

## 1 Introduction

Postoperative delirium (POD) is defined as an acute disorder of attention and cognition that occurs in the hospital up to 1 week post-procedure or until discharge (1, 2). The incidence of POD varies from 12 to 51% with different surgery types (3). POD is associated with an increase in adverse postoperative complications, delayed rehabilitation, and a higher mortality rate and imposes a heavy burden on the healthcare system (4, 5). A wide range of risk factors for POD has been suggested, including advanced age, pre-existing cognitive impairment, sleep disturbance, psychiatric disorders, and coexisting medical conditions (6–8).

Intraoperative hypotension (IOH) is commonly described as a decrease in the mean arterial pressure (MAP) or the systolic blood pressure (SBP) below a predefined threshold during surgery. However, there is no consensus definition, leading to substantial heterogeneity in its reporting across studies. This heterogeneity stems from variations in multiple definitional components: the blood pressure parameter (MAP vs. SBP), the threshold nature (absolute vs. relative), and often additional criteria such as minimum duration or number of episodes. Furthermore, studies quantify hypotension exposure using fundamentally different formats, primarily as dichotomous outcomes (IOH present/absent) or as continuous metrics (e.g., area under the curve). This lack of standardization directly impacts the reported incidence of IOH, which fluctuates widely between 6 and 44% (9–11) In several studies, IOH was thought to be associated with POD, one of the mechanisms of which was realized by reducing the cerebral blood infusion and oxygen supply (12, 13).

However, the association between IOH and POD remains inconclusive, as evidenced by conflicting conclusions from previous meta-analyses. These discrepant conclusions likely stem from limitations such as restricted search strategies, insufficient sample sizes, and crucially, a lack of systematic handling of the pronounced heterogeneity in IOH definitions across primary studies (14, 15). Therefore, we conducted a systematic review and meta-analysis and aimed to clarify whether IOH contributes to the development of POD.

## 2 Materials and methods

This meta-analysis was registered on PROSPERO (registration number: CRD42023424166) and performed according to the Preferred Reporting Items for Systematic Reviews and Meta-Analysis (PRISMA) guidelines (16).

### 2.1 Search strategy

Two authors (XW-Y and HL-Z) independently searched four databases (PubMed, EMBASE, Cochrane Library, and Web of Science) from database establishment to June 14, 2025. Search terms contained both Medical Subject Headings (MeSH) terms and free text to define exposure (IOH) and outcome (POD). The full search strategy is available in Supplementary Data.

### 2.2 Study selection and eligibility

Two authors (XW-Y and HL-Z) independently reviewed the identified studies. The full-text of potentially relevant articles was retrieved after screening titles and abstracts for eligibility. After the removal of duplicates, we screened the titles and abstracts for relevance and retrieved the accessible full text to identify the eligibility of studies for inclusion. Reviews, conference abstracts, letters, case series, and studies of pediatric surgery were all excluded. Disagreements were resolved by discussion with another author (Q-L).

### 2.3 Inclusion and exclusion

Inclusion criteria: (a) Design: Randomized controlled trials (RCTs) and observational studies; (b) Population: patients undergoing elective surgery under general anesthesia; (c) Intervention or exposure: different blood pressure groups or IOH with a clear definition; (d) Primary outcomes: POD evaluated with any proven and effective measuring tool. Exclusion criteria: (a) non-availability of full texts; (b) language other than English or Chinese.

### 2.4 Data extraction

Two authors (XW-Y and HL-Z) extracted data from each study, and conflicts were resolved by consensus with another author (Q-L) to ensure consistency and accuracy. The following information was extracted from each included study: study design, patient characteristics, type of surgery, definition of IOH, screening material of POD, timing of POD screening, definition of POD, and POD incidence.

To systematically manage the heterogeneity in the definition of IOH, we deconstructed its core component. The core components of any definition of IOH included the Blood Pressure Parameter (MAP or SBP) and the Threshold Nature (absolute threshold or relative threshold). A proportion of definitions intended to generate a dichotomous outcome incorporated additional criteria for dichotomization, such as stipulating a minimum duration or a minimum number of episodes below the threshold, as a requisite for confirming an IOH event.

Based on the format of the data presented for the IOH variable, studies were classified into two distinct groups. The first group, termed “Dichotomous Definitions,” utilized the core components to classify patients into “IOH present” or “IOH absent.” The second group, termed “Continuous Metrics,” quantified hypotension exposure as a continuous variable using distinct calculation methods, such as variably derived Area Under the Curve (AUC) or other composite measures. Owing to fundamental inconsistencies in the algorithms for these continuous metrics across studies—which introduced insurmountable methodological heterogeneity—studies in this category were included in the qualitative synthesis but excluded from the meta-analysis.

Finally, to ensure clarity and avoid double-counting in our synthesis, when a study reported multiple IOH definitions, a single ‘primary’ definition was selected for synthesis. The selection was based on a frequency analysis across all included studies: we first tabulated all unique definitions from studies that reported only one definition. For studies reporting multiple definitions, the definition that matched the most frequently occurring one in the aforementioned tabulation was selected as the primary definition. Similarly, multiple comparable groups within a single study were combined into a single group for analysis.

### 2.5 Risk of bias

Two authors (XW-Y and HL-Z) independently assessed the quality of included studies using the Newcastle-Ottawa Scale for observational studies (17) and the Cochrane risk-of-bias tool for RCTs (18).

### 2.6 Data synthesis

Systematic review and meta-analysis were performed using Review Manager 5.4.1. The analytical approach was determined by the classification of the IOH definition. For studies employing “Dichotomous Definitions,” data were extracted into 2 × 2 contingency tables to calculate the effect size. Conversely, studies utilizing “Continuous Metrics” for IOH were not included in the meta-analysis due to insurmountable methodological heterogeneity in pooling these measures. However, the findings from these studies were presented descriptively.

We maintained segregation between RCTs and observational studies in our meta-analysis due to fundamental methodological disparities and potential clinical heterogeneity across study designs. Risk ratio (RR) and odds ratio (OR) were calculated separately for RCTs and observational studies. A random-effect meta-analysis model was established to test the difference in incidence of POD between patients with or without IOH (19). The inverse variance method was used for weighing the studies. Between-study variance was estimated using the DerSimonian and Laird method. The I^2^ value was calculated to evaluate heterogeneity. An I^2^ value > 50% indicates substantial heterogeneity. Due to the limited number of available RCTs, both the subgroup analyses and meta-regression were restricted to observational studies to ensure robust and interpretable results. Subgroup analyses were performed to descriptively compare effect sizes across categories of IOH definition, screening material of POD, and region. To explore sources of heterogeneity, univariable meta-regression analyses were then applied using the restricted maximum-likelihood method to examine the relationship between these pre-specified covariates and the effect size; a multivariable meta-regression was not performed due to the limited number of studies available for each model, which would have resulted in an underpowered and statistically unstable analysis. Furthermore, sensitivity analysis was performed to assess the influence of individual studies on the pooled results by sequentially excluding each study. A funnel plot was used to assess the potential publication bias. Given the limited number of RCTs, which precludes reliable testing, Egger’s linear regression test was applied specifically to the pooled analysis of observational studies to statistically evaluate funnel plot asymmetry.

## 3 Results

### 3.1 Search results and study characteristics

**Figure 1:**
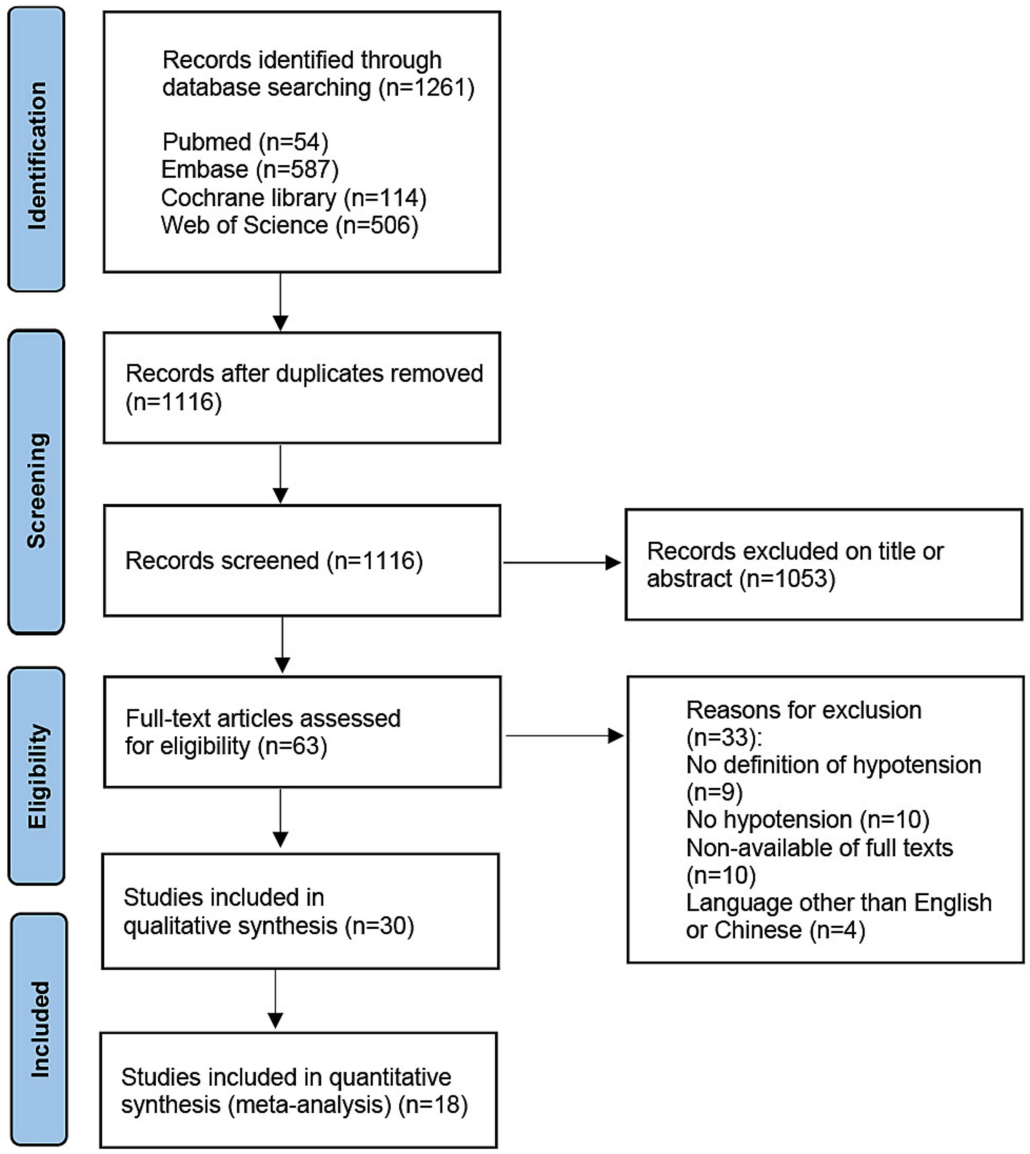
PRISMA study flow diagram.

The study selection process is presented in Figure 1. The initial search produced 1,261 articles. After 145 duplicate references were removed, a total of 1,116 articles were screened for eligibility. Sixty-three articles remained after excluding records according to title or abstract. After screening the full texts, 33 articles were excluded. As a result, a total of 30 articles with 372,158 patients were included in the systematic review, of which 18 articles with 322,879 patients were fit for the meta-analysis (9–11, 14, 15, 20–44).

The detailed characteristics of the 30 articles included are presented in Table 1. Overall, this review included five RCTs, 15 cohort studies, and 10 case–controlled studies with publication dates ranging from 1998 to 2025. The mean age ranged from 48.34 ± 12.87 years to 81.5 ± 5.5 years. Langer et al. (30) designed an RCT; however, since the outcome of interest was studied in a cohort design, it was classified as a cohort study.

**Table 1:** Study characteristics of included studies.

First author	Country	Study design	Sample size	Type of surgery	Incidence of delirium or brief description of results	
					In hypotensive group *n* (%)	In normotensive group *n* (%)
Studies included in the meta-analysis						
Marcantonio et al. (20)	United States	Case–control study	1,341 POD: 117 No POD: 1224	Noncardiac surgery	27 (8%)	90 (9%)
Patti et al. (21)	Italy	Case–control study	100 POD: 18 No POD: 82	Elective non-laparoscopic colorectal surgery for carcinoma	8 (44%)	10 (11%)
Tognoni et al. (22)	Italy	Case–control study	90 POD: 8 No POD: 82	Major (open) surgery: transvesical prostatectomy, radical retropubic prostatectomy, radical nephrectomy, radical cystectomy minor (endoscopic) surgery: trans-urethral resection of prostate that exceeded 60 min of surgical session	5 (20%)	3 (5%)
Yang et al. (11)	China	Case–control study	480 POD: 137 No POD: 343	Elective surgery	8 (28%)	129 (29%)
Guo et al. (23)	China	Case–control study	385 POD: 18 No POD: 82	Elective early escharotomy	44 (58%)	12 (4%)
Kim et al. (25)	Korea	Case–control study	318 POD: 19 No POD: 299	Total knee arthroplasty	10 (14%)	9 (4%)
Wu et al. (14)	China	Case–control study	343 POD: 103 No POD: 240	Elective cardiothoracic surgery using general anesthesia	57 (49%)	46 (20%)
Nakatani et al. (15)	Japan	Case–control study	324 POD: 26 No POD: 298	Elective transurethral resection of bladder tumor for bladder tumors	4 (8%)	22 (8%)
Lyu et al. (26)	China	Case–control study	583 POD: 106 No POD: 477	Intestinal obstruction or gastrointestinal perforation and underwent urgent surgical procedures	91 (22%)	15 (8%)
Hirsch et al. (9)	United States	Cohort study	540 IOH: 164 No IOH: 376	Non-cardiac surgery	MAP decrease 20%: 91 (34%) MAP decrease 30%: 55 (34%) MAP decrease 40%: 18 (30%) MAP < 50 mmHg: 8 (25%)	MAP decrease 20%: 87 (32%) MAP decrease 30%: 123 (33%) MAP decrease 40%: 160 (33%) MAP < 50 mmHg: 170 (33%)
Wang et al. (29)	China	Cohort study	323 IOH: 134 No IOH: 189	Laryngectomy	17 (13%)	11 (6%)
Wachtendorf et al. (10)	United States	Cohort study	316,717 No IOH: 176670 Short IOH: 131922 Prolonged IOH: 8125	Noncardiac surgery under general anesthesia	Short (< 15 min) duration of MAP < 55 mmHg: 1176 (0.9%) Prolonged (≥ 15 min) duration of MAP < 55 mmHg: 112 (1.4%)	No intraoperative hypotension: 895(0.5%)
Wang et al. (39)	China	Cohort study	428 IOH: 166 No IOH: 262	Laryngectomy under general anesthesia	38 (23%)	39 (15%)
Williams-Russo et al. (40)	United States	Randomized controlled trial	235 Low-level MAP: 117 High-level MAP: 118	Elective unilateral primary total hip replacement	11 (9%)	5 (4%)
Siepe et al. (41)	Germany	Randomized controlled trial	92 Low-level MAP: 48 High-level MAP: 44	Elective or urgent coronary artery bypass graft surgery	6 (13%)	0 (0%)
Xu et al. (42)	China	Randomized controlled trial	150 Low-level MAP: 50 High-level MAP: 100	Elective hip replacement	17 (34%)	15 (15%)
Hu et al. (43)	China	Randomized controlled trial	322 Low-level MAP: 143 High-level MAP: 155	Non-cardiothoracic surgery with general anesthesia	36 (25%)	18 (12%)
Zhang et al. (44)	China	Randomized controlled trial	108 Low-level MAP: 55 High-level MAP: 53	Gastrointestinal laparoscopic surgery	18 (33%)	15 (28%)
Studies not included in the meta-analysis						
Jiang et al. (24)	China	Case–control study	451 POD: 42 No POD: 409	Spinal surgery	Incidence of intraoperative hypotension was shown to be related to postoperative delirium (1.5 ± 0.6 times and 0.8 ± 0.4 times for delirious and non-delirious patients, respectively).	
Wesselink et al. (27)	Holland	Cohort study	734	Cardiac surgery requiring cardiopulmonary bypass	After adjusting for confounding and multiple testing, there were no significant associations between IOH based on any of the definitions and delirium	
Tobar et al. (28)	Chile	Cohort study	28	Major open colon surgery	AUC of absolute minimum MAP values below 50 and 60 mmHg did not found to have a significant difference between patients without delirium and patients with delirium	
Langer et al. (30)	Italy	Randomized controlled trial	101	Elective non-cardiac surgery	No correlation was found between intraoperative hypotension, expressed as hypotension index, and delirium (*p* = 0.19)	
Maheshwari et al. (31)	United States	Cohort study	1,083	Noncardiac surgery	One mm Hg increase in TWA MAP <65 mm Hg was significantly associated with an increased cause-specific hazard of delirium (HR, 1.11; 95% CI, 1.03–1.20; *p* = 0.009)	
Wesselink et al. (32)	Holland	Cohort study	675	Transcatheter aortic valve replacement	Did not find a statistically significant association between IOH for any threshold and the occurrence of POD after transcatheter aortic valve replacement	
Narayanan et al. (33)	India	Cohort study	50	Cancer surgery	There was no statistically significant association between IOH (decrease of MAP or SBP > 20% from baseline and a MAP or SBP decrease of >40% from baseline) and POD	
Ushio et al. (34)	Japan	Cohort study	503	Valvular surgery that required cardiopulmonary bypass	There was no significant difference in any thresholds of IOH in the period of during surgery	
Duan et al. (35)	China	Cohort study	605 No IOH: 202 Short IOH: 186 Long IOH: 217	Thoracic surgery or orthopedic surgery under general anesthesia	Short duration of hypotension (< 5 min) was not associated with POD (adjusted OR 1.18; 95% CI: 0.56–2.50, *p* = 0.671), while long duration of hypotension (≥ 5 min) increased the risk of POD (adjusted OR 3.93; 95% CI: 2.07–7.45, *p* < 0.001).	
Mohr et al. (36)	Germany	Cohort study	31,315	Elective cardiac surgery with and without cardiopulmonary bypass	After risk adjustment, there was still a significant association between the frequency of IOH episodes and the occurrence of postoperative delirium (OR 1.02, 95% CI 1.003–1.03, *p* < 0.001).	
Zarour et al. (37)	Israel	Cohort study	2,352	Elective noncardiac surgery with general anesthesia	In the univariate analysis, incidence of postoperative delirium did not differ between AUC quartile groups (first quartile, 13.2%; second quartile, 14.0%; third quartile, 13.6%; and fourth quartile, 15.0%, *p* = 0.82). After adjustment for potential confounding variables, intraoperative hypotension was also not associated with postoperative delirium.	
Singh et al. (38)	United States	Cohort study	11,382	Aortic, mitral, or tricuspid valve repair/replacement, coronary artery bypass grafting, ascending aorta replacement, or a combination of these procedures	We did not have sufficient evidence to reject the null hypothesis of no association between TWA-MAP <60 mmHg and POD during any time period. After adjusting for multiple testing, none of the likelihood ratio tests was statistically significant	

AUC, Area under the curve; IOH, Intraoperative hypotension; MAP, Mean arterial pressure; OR, Odds ratio; POD, Postoperative delirium; SBP, Systolic blood pressure; TWA, Time-weighted average.

The definitions of IOH and POD are classified and summarized in Table 2. Following our pre-specified classification criteria, the definitions of IOH from the included studies were systematically categorized. Core components of the definitions, namely the blood pressure parameter and threshold nature, were analyzed across all studies. Among the 30 included studies, 25 used an absolute threshold, with the most frequently used definition (employed by nine studies) being a MAP less than 60 mmHg. Ten studies utilized a relative threshold, the most common of which (in five studies) was a MAP decline to less than 70% of the preoperative baseline. Based on how these components were applied for data presentation, studies were grouped accordingly. In five RCTs and seventeen observational studies, IOH was defined using “Dichotomous Definitions.” Furthermore, four of these studies incorporated additional criteria for dichotomization, requiring that blood pressure remain below the threshold for a defined duration or occur in a specified number of episodes to confirm an IOH event. The remaining eight observational studies quantified hypotension exposure using “Continuous Metrics,” the majority of which employed variably calculated AUC. The screening tools for POD varied between studies. The Confusion Assessment Method (CAM) and its intensive care unit variant (CAM-ICU) were used to screen for POD in 14 studies and seven studies, respectively. The Mini-Mental State Examination (MMSE) or International Classification of Diseases (ICD) codes were applied in the other seven studies. Time points used to evaluate cognitive function also varied between studies. Only eight studies performed POD screening lasting up to 7 days after surgery.

**Table 2:** Definition of intraoperative hypotension and postoperative delirium in the included studies.

First author	Core components of the definition of IOH				Additional criteria for dichotomization^a^	Timing of POD screening	Screening material of POD
	Absolute threshold and MAP	Absolute threshold and SBP	Relative threshold and MAP	Relative threshold and SBP			
Marcantonio et al. (20)		< 90 mmHg		< 66% of preoperative baseline	Not reported	On postoperative days 2–5, or until the day before discharge	CAM, The Chart/Nursing Intensity Index criteria
Patti et al. (21)	≤ 60 mmHg				Not reported	Until discharge	CAM
Tognoni et al. (22)		< 90 mmHg			Not reported	A week	CAM
Yang et al. (11)			< 70% of preoperative baseline		Not reported	A week	CAM
Guo et al. (23)	< 55 mmHg				Not reported	5 days	CAM
Jiang et al. (24)		< 80 mmHg			Not reported	Not reported	The clinical features of postoperative delirium, as Inouye(2006) summarized
Kim et al. (25)		< 90 mmHg			Any documented occurs 3 times	Not reported	DSM-5
Wu et al. (14)	< 60 mmHg				Not reported	A week	CAM-ICU
Nakatani et al. (15)	< 60 mmHg				Not reported	A week	a chart-based method
Lyu et al. (26)		< 90 mmHg			Not reported	7 postoperative days	DSM-V, 2013
Hirsch et al. (9)	< 50 mmHg		< 80% of the preoperative baseline or < 70% of the preoperative baseline or < 60% of preoperative baseline	< 80% of the preoperative baseline or < 70% of the preoperative baseline or < 60% of preoperative baseline	Not reported	2 days	CAM
Wesselink et al. (27)	< 60 mmHg or < 50 mmHg		< 70% of the preoperative baseline or < 60% of preoperative baseline		Continuous Metrics	4 days	CAM, CAM-ICU, medical records
Tobar et al. (28)	< 60 mmHg or < 50 mmHg		< 80% of the preoperative baseline or < 70% of the preoperative baseline or < 60% of preoperative baseline		Continuous metrics	A week	CAM
Wang et al. (29)			< 70% of preoperative baseline		last at least 30 min	6 days	CAM
Langer et al. (30)			< 90% of preoperative baseline		Continuous metrics	Not reported	CAM-ICU
Maheshwari et al. (31)	< 65 mmHg				Continuous metrics	5 days	CAM-ICU, Richmond Agitation-Sedation Scale
Wesselink et al. (32)	< 100 mmHg or < 90 mmHg or < 80 mmHg or < 70 mmHg or < 60 mmHg				Continuous metrics	Not reported	DSM-5, delirium observational score
Narayanan et al. (33)	< 60 mmHg		< 80% of preoperative baseline or < 60% of preoperative baseline	< 80% of preoperative baseline or < 60% of preoperative baseline	Not reported	3 days	The short-CAM
Ushio et al. (34)	< 85 mmHg or < 80 mmHg or < 75 mmHg or < 70 mmHg or < 65 mmHg or < 60 mmHg or < 55 mmHg or < 50 mmHg				Continuous metrics	A week	The intensive care delirium screening checklist
Wachtendorf et al. (10)	< 55 mmHg				Short intraoperative hypotension: <15 min prolonged intraoperative hypotension: > = 15 min	30 days	International classification of diseases, ninth/tenth revision (ICD-9/10)
Duan et al. (35)	≤ 60 mmHg				Short duration of hypotension: <5 min long duration of hypotension: > = 5 min	The first 3 postoperative days	CAM or CAM-ICU
Mohr et al. (36)	< 60 mmHg				Last for >2 min	In every shift	Nursing delirium screening scale tools; the international classification of diseases, tenth revision, and the diagnostic and statistical manual of mental disorders IV/V criteria.
Zarour et al. (37)	< 65 mmHg				Continuous metrics	1 h after admission to the post-anesthesia care unit (PACU), before PACU discharge, and on postoperative days 1 and 2	The 4 A’s tests and the validated chart-based delirium identification instrument (CHART-DEL) method
Singh et al. (38)	< 60 mmHg				Continuous metrics	Between 12 and 96 h after surgery	CAM, CAM-ICU, or brief CAM (bCAM) tools
Wang et al. (39)			< 70% of preoperative baseline		Lasting for at least 30 min	The first 5 days after surgery	CAM
Williams-Russo et al. (40)	Lower blood pressure group in RCT: 45–55 mmHg				RCT	Not reported	DSM-III-R
Siepe et al. (41)	Lower blood pressure group in RCT: 60–70 mmHg				RCT	2 days	MMSE
Xu et al. (42)			Lower blood pressure group in RCT: 80–90% of preoperative baseline or 90–100% of preoperative baseline		RCT	3 days	CAM-CR
Hu et al. (43)	Lower blood pressure group in RCT: 60–70 mmHg				RCT	A week	CAM-ICU
Zhang et al. (44)	Lower blood pressure group in RCT: 65–85 mmHg				RCT	5 days	CAM-CR

CAM, Confusion Assessment Method; CAM-CR, Confusion Assessment Method -Chinese Reversion; CAM-ICU, Confusion Assessment Method for the Intensive Care Unit; DSM-III-R, Revised edition of Diagnostic and Statistical Manual of Mental Disorders, third edition; DSM-5, Diagnostic and statistical manual of mental disorders, 5th edition; MAP, Mean arterial pressure; MMSE, Mini-Mental State Examination; RCT, Randomized controlled trial; SBP, Systolic blood pressure.

a Not report indicates that the original publication did not specify whether additional criteria (e.g., a minimum duration) were required for an IOH event.

Fifteen out of 30 studies found no statistically significant differences in the incidence of POD between hypotensive and normotensive patients, and these 15 studies comprised three RCTs and twelve observational studies. Seven studies investigated the association between IOH and POD in cardiac surgeries, and three of these found a significant difference. However, a formal statistical comparison between cardiac and non-cardiac surgeries was precluded by both methodological and design constraints. Among the seven cardiac surgery studies, the majority (four using Continuous Metrics and one cohort with unextractable data) were inherently excluded from the planned observational study-based subgroup analysis. This left only one eligible observational study (a case–control study) for the cardiac surgery group. A comparison based on a single study is statistically untenable, thus preventing a valid subgroup analysis. The POD incidence was reported in 18 studies out of the 22 studies that described IOH using “Dichotomous Definitions,” ranging from 0.9 to 58% in the hypotensive groups and 0 to 33% in the normotensive groups. Of the eight studies that quantified hypotension exposure using “Continuous Metrics,” only Maheshwari et al. found that IOH was associated with POD; however, in the other seven studies, IOH was not related to POD.

### 3.2 Meta-analysis

**Figure 2:**
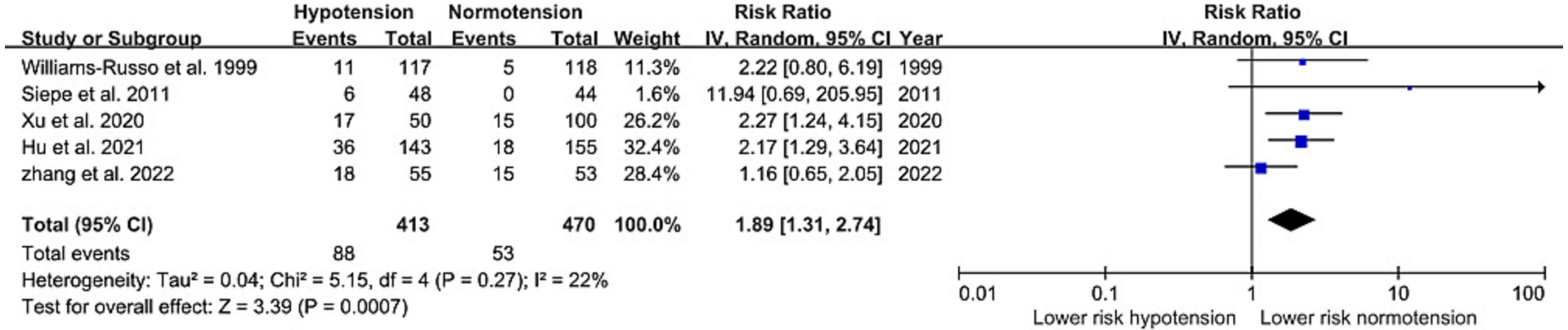
Primary outcome (RCTs)—forest plot showing the risk ratios for the association of intraoperative hypotension with postoperative delirium.

**Figure 3:**
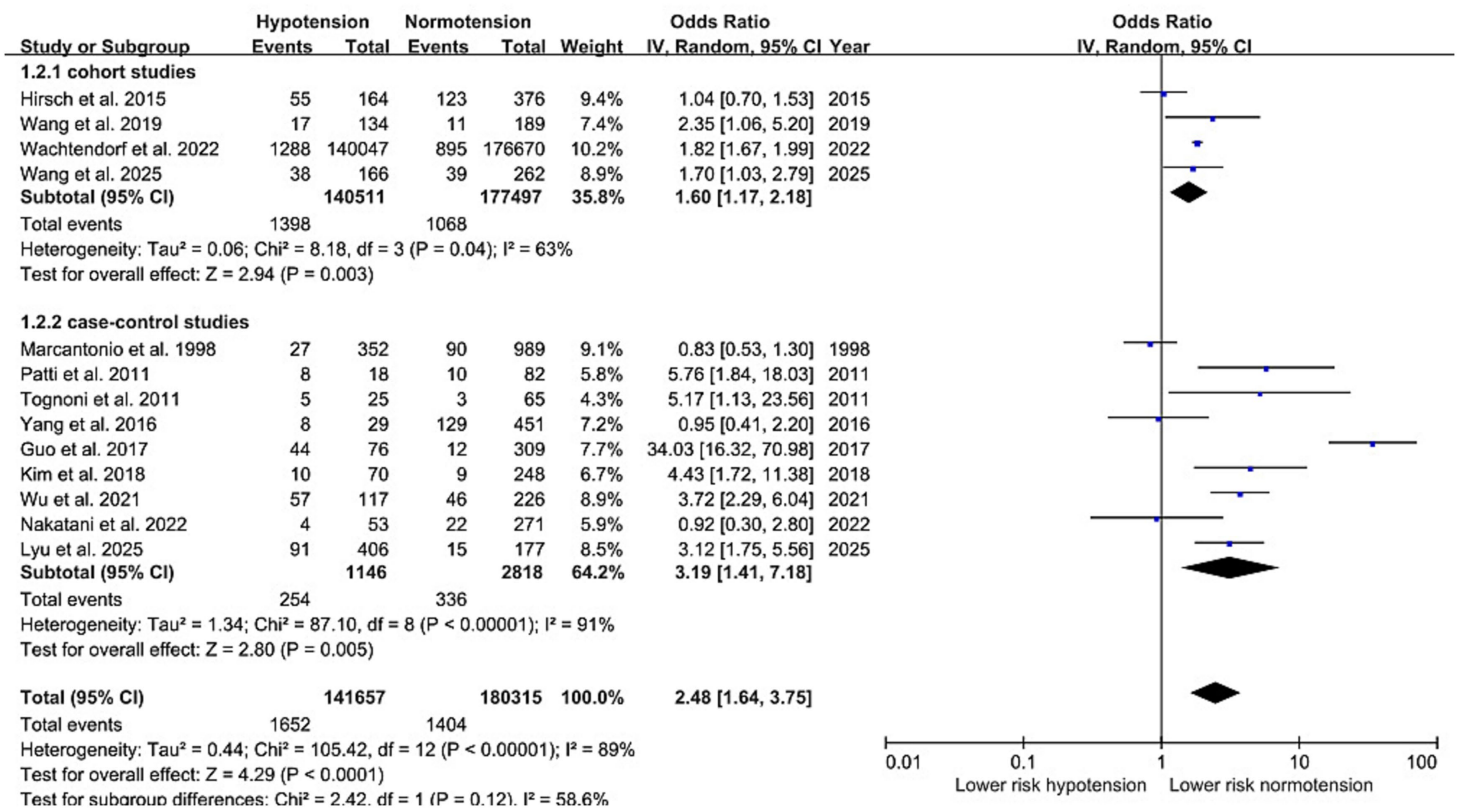
Primary outcome (observational studies)—forest plot showing the odds ratios for the association of intraoperative hypotension with postoperative delirium.

As shown in Figures 2, 3, IOH was significantly associated with an elevated risk of POD in both RCTs (RR: 1.89, 95%CI: 1.31–2.74) and observational studies (OR: 2.48, 95%CI: 1.64–3.75). Heterogeneity between observational studies was high (I^2^ = 89%), whereas it was low (I^2^ = 22%) among RCTs.

**Figure 4:**
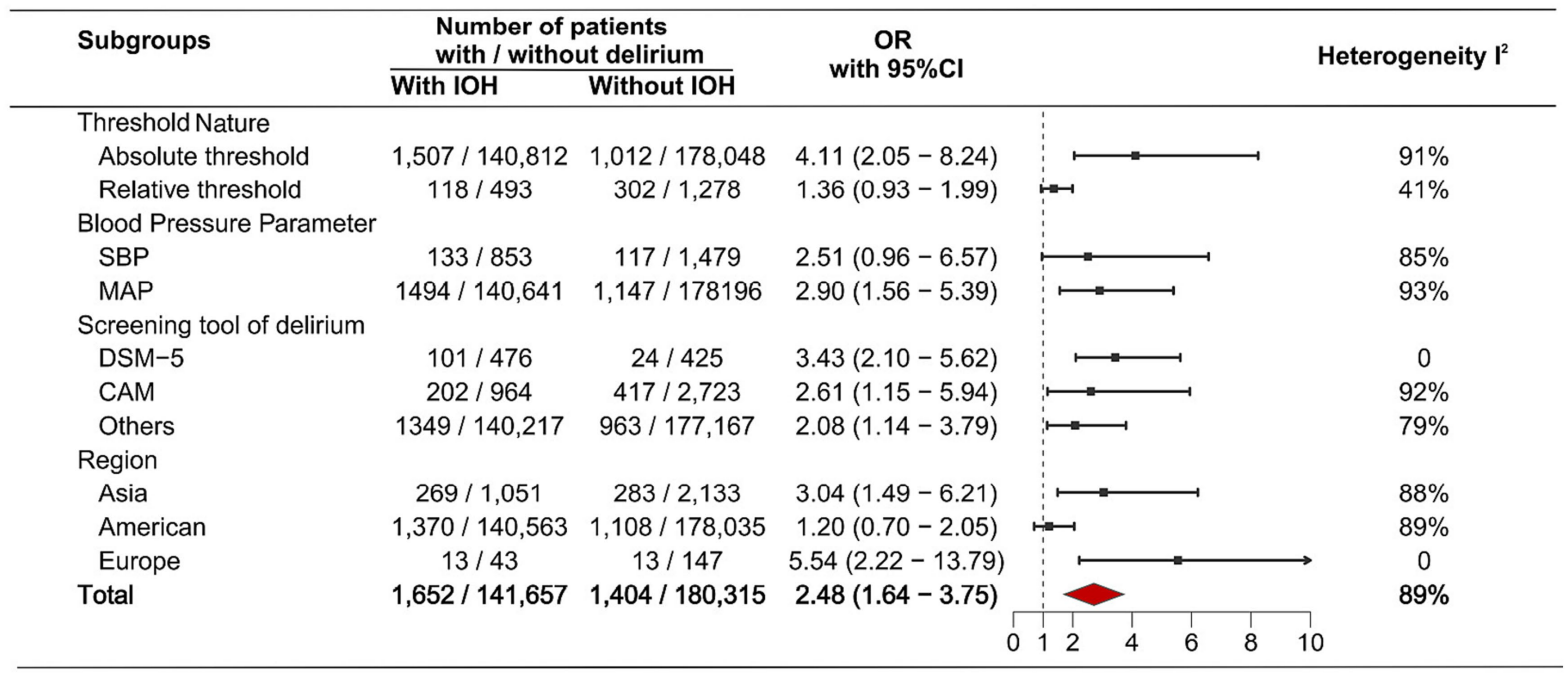
Subgroup analysis (observational studies)—forest plot of odds ratios for the association between intraoperative hypotension and postoperative delirium. Results are stratified by the definition of intraoperative hypotension (Threshold Nature, Blood Pressure Parameter), the Screening tool of delirium, and Region. CAM, Confusion Assessment Method; DSM-5, Diagnostic and statistical manual of mental disorders, 5th edition; IOH, Intraoperative hypotension; MAP, Mean arterial pressure; SBP, Systolic blood pressure.

Figure 4 shows the findings of the subgroup meta-analysis of observational studies based on various factors. In the subgroup analysis stratified by the Threshold Nature of the IOH definition, a significant association with a higher risk of POD was observed for absolute thresholds (OR: 4.11, 95% CI: 2.05–8.24). Conversely, no significant association was identified for relative thresholds (OR: 1.36, 95% CI: 0.93–1.99). When stratified by the Blood Pressure Parameter used in the definition, a significant association was observed for definitions based on MAP (OR: 2.90, 95% CI: 1.56–5.39), whereas the association for definitions based on SBP did not reach statistical significance (OR: 2.51, 95% CI: 0.96–6.57). Additionally, IOH was associated with a higher risk of POD in the subgroup meta-analysis using the POD screening tool. In the subgroup meta-analysis based on region, IOH was related to an increased risk of POD in both Asia (OR: 3.04, 95%CI: 1.49–6.21) and Europe (OR: 5.54, 95%CI: 2.22–13.79) studies, but IOH showed no significant association with POD (OR: 1.20, 95%CI: 0.70–2.05) in studies of America.

To investigate potential sources of high heterogeneity, we performed a univariate meta-regression using the same variables as in the subgroup analyses. As shown in Supplementary Table S1, the association between IOH and POD was notably affected by the moderator Threshold Nature, substantially reducing the tau value (from 0.9526 to 0.8069) and the I^2^ statistic (from 94.14 to 88.94%), suggesting that this moderator explains a portion of the observed heterogeneity. Furthermore, as shown in Supplementary Table S2, this variable significantly modified the adjusted overall estimate (OR: 2.86, *p* = 0.048), indicating it is an important effect modifier. Conversely, other moderators, including Blood Pressure Parameter, region, study type, and delirium assessment tool, did not significantly explain heterogeneity or alter the effect size, with *p*-values for the moderator tests all greater than 0.05.

### 3.3 Sensitivity analysis and publication bias

**Figure 5:**
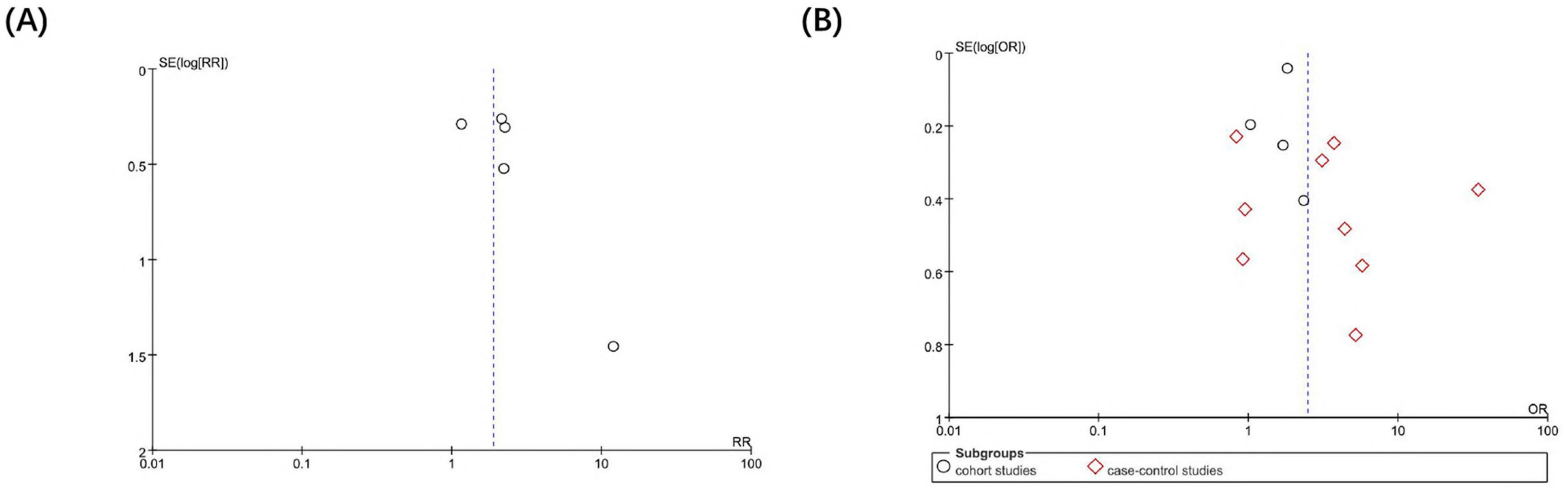
Funnel plot for RCTs and observational studies. **(A)** Funnel plot for RCTs. **(B)** Funnel plot for observational studies.

Sensitivity analysis was performed to explore the impact of individual studies on the pooled results. The sequential exclusion of any of the included studies did not change the stability and reliability of the results. Visual inspection of the funnel plots in Figure 5 showed a generally symmetrical distribution across both RCTs and observational studies, with larger and smaller studies reporting both negative and positive results. Given the limited number of RCTs, formal statistical testing for publication bias was not performed. For observational studies, publication bias was further assessed using Egger’s linear regression test, which indicated no statistically significant asymmetry (*p* = 0.37). Taken together, these assessments suggest that the overall findings are unlikely to be substantially influenced by publication bias.

### 3.4 Quality assessment

**Figure 6:**
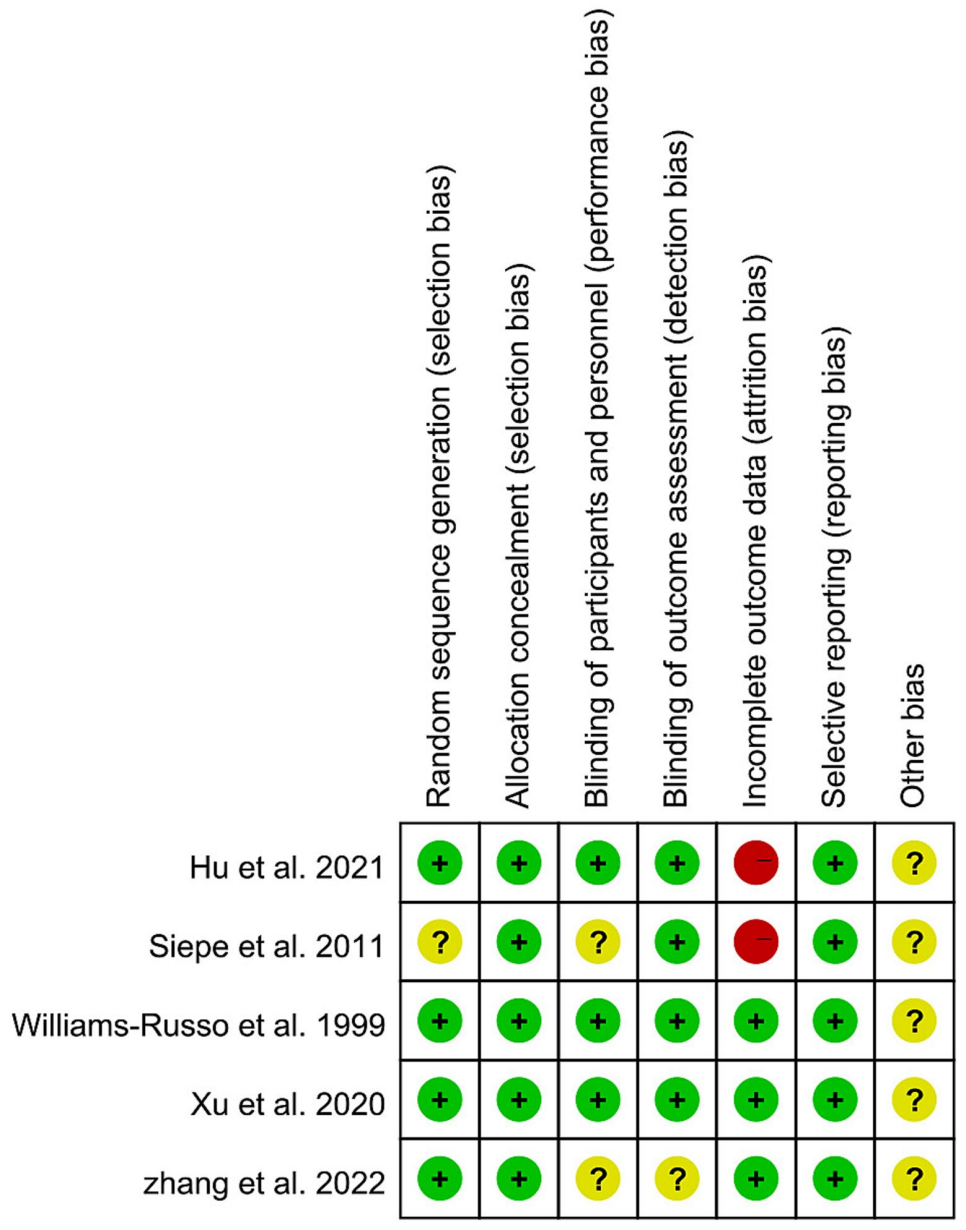
Risk of bias summary: authors’ judgement on risk of bias for included RCTs. Assessed using the Cochrane Collaboration’s Risk of Bias Tool. Green: low risk/Yellow: unclear risk/Red: high risk.

The quality of the included studies is shown in Figure 6 and Tables 3, 4. A qualitative summary of the main concerns is as follows: For RCTs, the overall risk of bias was low. The primary limitation was the inherent risk of performance bias, given the impossibility of blinding clinicians to intraoperative blood pressure management. For cohort studies, common methodological concerns included insufficient follow-up duration for the outcome and questions regarding the representativeness of the exposed cohort in some studies. The selection and comparability of controls in case–control studies were generally well addressed.

**Table 3:** Risk of bias summary: authors’ judgement on risk of bias for included cohort studies.^a^

Study	Selection				Comparability	Outcome			Score^b^
	S1	S2	S3	S4		O1	O2	O3	
Hirsch et al. (9)	0	1	1	0	0	1	0	1	4
Wesselink et al. (27)	0	1	1	0	2	1	0	1	6
Tobar et al. (28)	0	1	1	1	0	1	1	1	6
Wang et al. (29)	0	1	0	1	2	1	1	1	7
Langer et al. (30)	0	1	1	1	0	1	0	1	5
Maheshwari et al. (31)	0	1	0	1	2	1	0	1	6
Wesselink et al. (32)	0	1	1	0	2	1	0	0	5
Narayanan et al. (33)	0	1	1	1	0	1	0	1	5
Ushio et al. (34)	0	1	1	0	2	1	0	0	5
Wachtendorf et al. (10)	0	1	0	1	2	1	1	1	7
Duan et al. (35)	0	1	1	1	2	1	0	1	7
Mohr et al. (36)	0	1	1	0	2	1	0	0	5
Zarour et al. (37)	0	1	1	1	2	1	0	0	6
Singh et al. (38)	0	1	1	0	2	1	0	1	6
Wang et al. (39)	0	1	1	1	2	1	1	1	8

a Assessed using the Newcastle-Ottawa Quality Assessment Scale.

b Higher quality studies are reflected by a higher score.

**Table 4:** Risk of bias summary: authors’ judgement on risk of bias for included case–control studies.^a^

Study	Selection				Comparability	Exposure			Score^b^
	S1	S2	S3	S4		E1	E2	E3	
Marcantonio et al. (20)	1	1	1	1	0	1	1	1	7
Patti et al. (21)	1	0	1	1	2	0	1	1	7
Tognoni et al. (22)	1	0	1	1	0	1	1	1	6
Yang et al. (11)	1	1	1	1	2	1	1	1	9
Guo et al. (23)	1	0	1	1	2	0	1	1	7
Jiang et al. (24)	1	0	1	1	2	1	1	1	8
Kim et al. (25)	1	0	1	1	2	1	1	1	8
Wu et al. (14)	1	1	1	1	2	0	1	1	8
Nakatani et al. (15)	1	0	1	1	2	0	1	1	7
Lyu et al. (26)	1	0	1	1	2	1	1	1	8

a Assessed using the Newcastle-Ottawa Quality Assessment Scale.

b Higher quality studies are reflected by a higher score.

## 4 Discussion

The quantitative analysis performed on observational studies and RCTs showed that IOH was associated with an increased risk of POD. A critical finding emerged from further investigation: the Threshold Nature of the IOH definition was identified as a pivotal factor. Subgroup analysis revealed a strong and significant association for absolute threshold, but not for relative threshold. This striking discrepancy was confirmed by meta-regression, which established the Threshold Nature as a significant source of heterogeneity and a key effect modifier.

Previous studies have found that IOH is strongly associated with myocardial injury, myocardial infarction, renal injury, and death (12). Organ hypoperfusion, including the heart, kidneys, and brain, might be the potential cause of these postoperative complications. However, so far, some evidence has revealed that postoperative neurological complications might not be associated with brain hypoperfusion. The study conducted by Feng et al. (45) suggested no significant correlation between IOH and the incidence of POD based on only two RCTs. Furthermore, Wijnberge et al. (46) found that IOH was not associated with delirium in a subgroup meta-analysis comprising three cohort studies. However, in a recent meta-analysis by Cai et al. (12), found that IOH was associated with POD in non-cardiac surgery based on the results synthesized by two RCTs and 15 cohort studies. We conducted a comprehensive search, including the latest literature. The conclusions of the aforementioned studies were limited by a combination of factors, including small sample size, inadequate search strategy, and a predominance of observational studies. Thus, we designed this meta-analysis and found that IOH was associated with the increased risk of POD in both RCTs and observational studies.

Studies that defined IOH by absolute threshold found a stronger association than studies that defined IOH by relative threshold. Besides, the comparison of studies defining IOH by MAP to studies defining IOH by SBP was consistent with the above. This pattern can be explained mechanistically by the inherent difference in these definitional criteria. For a substantial subset of patients, particularly those with hypertension, a relative threshold corresponds to a higher absolute pressure than an absolute threshold. Similarly, the SBP threshold is physiologically higher than the MAP threshold. This is empirically supported by Wickham et al., who reported a higher incidence of IOH when using relative or SBP-based definitions, indicating that for many individuals, these criteria identify a less severe state of hypotension (47). Consequently, definitions using absolute or MAP thresholds serve as more specific markers of severe hypotension, whereas those using relative or SBP thresholds capture a broader, heterogeneous group with a diluted association to POD. Given the stronger and more consistent association observed with absolute MAP thresholds, our findings support the use of standardized definitions centered on an absolute MAP value (e.g., < 60 mmHg) in future studies of IOH and POD. This approach is recommended to reduce definitional heterogeneity and facilitate comparability across studies. Ultimately, to definitively determine the optimal threshold for clinical guidance, a definitive large-scale RCT is required to directly compare the effects of multiple candidate MAP thresholds on POD incidence.

The screening material used in our included studies for POD is quite different. Diagnostic and Statistical Manual of Mental Disorders, 5th edition (DSM-5) criteria, the gold standard for diagnosing delirium, were used in only two of the quantitative studies because they are difficult for non-psychiatrists to apply in clinical practice. Both of these studies supported the finding that IOH is associated with POD. Eight studies included in our analysis used CAM as a POD identification instrument, and three of these eight studies found that IOH was not associated with POD. Different POD screening tools identify delirium somewhat differently and assess different domains. Thus, different POD screening tools cause the reported incidence of POD to fluctuate across included studies, which is one reason why a consensus on the relationship between IOH and POD has not been reached.

Cerebral hypotension may be the most plausible mechanism by which IOH could result in POD. A reduction in cerebral blood flow can increase the probability of POD (48). The cerebral autoregulation system maintains stable cerebral blood flow over a relatively wide range as cerebral perfusion pressure varies. A sufficient decrease in arterial blood pressure could reach the lower boundary of cerebral autoregulation and result in a low cerebral blood flow (13). Different individuals may have different upper and lower limits of autoregulation. Besides, the limits of autoregulation are dynamic because of the multiple factors that influence it (49–51). These factors might also be a possible reason for the different results on the relationship between IOH and POD. Chen et al. found cerebral oximetry index-guided blood pressure management during CPB was associated with a reduced incidence and severity of POD following acute type A aortic dissection surgery (52). Their study suggested that monitoring the cerebral oximetry index might help to identify the patient-specific optimal blood pressure ranges that maintain adequate cerebral perfusion. Yamanoi et al. (53) found that a prolonged double-low period, defined as a bispectral index < 45 and a MAP <75 mmHg during general anesthesia, was independently associated with an increased incidence of POD in surgical ICU patients. Therefore, combining multimodal biomarkers might improve the individualized determination of cerebral autoregulation limits.

While cerebral hypoperfusion due to IOH represents one plausible mechanism, as discussed in the preceding paragraph, the recently proposed ‘Protective Hemodynamics’ strategy offers a complementary, hypothesis-generating perspective on the potential mechanisms underlying our results. This alternative view posits that hypotension may serve primarily as a marker of patients who will develop adverse events, rather than the direct cause. According to this paradigm, vasopressors administered to correct hypotension might induce excessive vasoconstriction in the splanchnic and cerebral vasculature, potentially leading to impaired organ perfusion and injury (54). Although RCTs included in our analysis support the association between IOH and POD, the limited number of available RCTs precludes definitive conclusions regarding causality. We cannot rule out the possibility that the aforementioned mechanism underlies our findings. Consequently, the primary clinical implication of our study, and a central question for future research, is to determine which therapeutic strategy is paramount for improving patient outcomes: rigorously avoiding hypotension per se, or minimizing exposure to vasopressors to reduce their potential detrimental effects. It is crucial to recognize that these two goals are not inherently mutually exclusive. Addressing this dilemma requires innovative clinical trial designs. Frameworks such as the C.L.E.A.R. approach, which operationalizes the principles of protective hemodynamics to simultaneously prevent profound hypotension and restrict vasopressor use, provide a conceptual basis for such future investigations (54).

This study has several limitations that should be considered when interpreting the findings. First, substantial heterogeneity was observed, particularly among the observational studies, which may limit the generalizability of the pooled results. This heterogeneity primarily stems from several methodological variations, primarily in the definitions of key variables. Specifically regarding IOH, the definitions varied in their core components, including the Blood Pressure Parameter and the Threshold Nature. Furthermore, among studies employing “Dichotomous Definitions,” there was inconsistency in the application of additional criteria for dichotomization. This methodological heterogeneity may reduce the persuasiveness of the pooled results, as differing definitions could influence the observed strength and direction of the association. Regarding POD assessment, it was not uniform across studies, employing different screening tools and assessment timings, which together could lead to outcome misclassification. Although a shorter screening window is pragmatically easier and may capture most episodes, it might not fully represent the entire postoperative course. In addition to the methodological heterogeneity discussed above, other unmeasured factors, such as variations in surgical populations, specific anesthesia protocols, and patient comorbidities, may have also contributed to the observed heterogeneity but could not be quantitatively assessed due to inconsistent reporting across the included studies. Finally, the current evidence base has constraints. The number of RCTs was limited, and most data were derived from observational studies that are susceptible to residual confounding. Furthermore, among the studies that quantified hypotension exposure using “Continuous Metrics,” the specific calculation methods were inconsistent across studies. Therefore, it is not currently possible to definitively establish a precise exposure-response relationship between the duration or depth of hypotension and the risk of POD based on the available evidence.

## 5 Conclusion

Our systematic review and meta-analysis found that IOH was statistically significantly associated with a higher risk of POD. However, the meta-analysis was limited by the numerous methodological differences among the included studies. Future research conducted in a standardized manner is needed to increase generalizability and facilitate easier interpretation of study results.

## Data Availability

The original contributions presented in the study are included in the article/Supplementary material, further inquiries can be directed to the corresponding authors.
