# Supplementary material for: The effect of intraoperative hypotension on postoperative delirium: a meta-analysis and systematic review
Source: Front Med (Lausanne). 2026 Jan 6;12:1690490. doi: 10.3389/fmed.2025.1690490 (PMC12816188; doi:10.3389/fmed.2025.1690490)
Supplement: Supplementary file 1 [file Supplementary_file_1.docx]

**The effect of intraoperative hypotension on postoperative delirium: a meta-analysis and systematic review**

**Supplementary data**

**Xiaowei Yin, Huolin Zeng, Qian Li, Qian Li, Hui Yang, Jin Liu**

**Contents**

**No Item page**

**1 PRISMA 2020 checklist 2**

**2 Search strategy 7**

**3 Table 9**

**PRISMA 2020 checklist**

| **Section and Topic** | **Item #** | **Checklist item** | **Location where item is reported** |
| --- | --- | --- | --- |
| **TITLE** | | |  |
| Title | 1 | Identify the report as a systematic review. | Page 1 |
| **ABSTRACT** | | |  |
| Abstract | 2 | See the PRISMA 2020 for Abstracts checklist. | Page 1,2 |
| **INTRODUCTION** | | |  |
| Rationale | 3 | Describe the rationale for the review in the context of existing knowledge. | Page 2,3 |
| Objectives | 4 | Provide an explicit statement of the objective(s) or question(s) the review addresses. | Page 3 |
| **METHODS** | | |  |
| Eligibility criteria | 5 | Specify the inclusion and exclusion criteria for the review and how studies were grouped for the syntheses. | Page 4 |
| Information sources | 6 | Specify all databases, registers, websites, organisations, reference lists and other sources searched or consulted to identify studies. Specify the date when each source was last searched or consulted. | Page 4 |
| Search strategy | 7 | Present the full search strategies for all databases, registers and websites, including any filters and limits used. | Page 4 |
| Selection process | 8 | Specify the methods used to decide whether a study met the inclusion criteria of the review, including how many reviewers screened each record and each report retrieved, whether they worked independently, and if applicable, details of automation tools used in the process. | Page 4 |
| Data collection process | 9 | Specify the methods used to collect data from reports, including how many reviewers collected data from each report, whether they worked independently, any processes for obtaining or confirming data from study investigators, and if applicable, details of automation tools used in the process. | Page 4 |
| Data items | 10a | List and define all outcomes for which data were sought. Specify whether all results that were compatible with each outcome domain in each study were sought (e.g. for all measures, time points, analyses), and if not, the methods used to decide which results to collect. | Page 4 |
|  | 10b | List and define all other variables for which data were sought (e.g. participant and intervention characteristics, funding sources). Describe any assumptions made about any missing or unclear information. | Page 4 |
| Study risk of bias assessment | 11 | Specify the methods used to assess risk of bias in the included studies, including details of the tool(s) used, how many reviewers assessed each study and whether they worked independently, and if applicable, details of automation tools used in the process. | Page 5 |
| Effect measures | 12 | Specify for each outcome the effect measure(s) (e.g. risk ratio, mean difference) used in the synthesis or presentation of results. | Page 5 |
| Synthesis methods | 13a | Describe the processes used to decide which studies were eligible for each synthesis (e.g. tabulating the study intervention characteristics and comparing against the planned groups for each synthesis (item #5)). | Page 5 |
|  | 13b | Describe any methods required to prepare the data for presentation or synthesis, such as handling of missing summary statistics, or data conversions. | Page 5 |
|  | 13c | Describe any methods used to tabulate or visually display results of individual studies and syntheses. | Page 5 |
|  | 13d | Describe any methods used to synthesize results and provide a rationale for the choice(s). If meta-analysis was performed, describe the model(s), method(s) to identify the presence and extent of statistical heterogeneity, and software package(s) used. | Page 5 |
|  | 13e | Describe any methods used to explore possible causes of heterogeneity among study results (e.g. subgroup analysis, meta-regression). | Page 5 |
|  | 13f | Describe any sensitivity analyses conducted to assess robustness of the synthesized results. | Page 5,6 |
| Reporting bias assessment | 14 | Describe any methods used to assess risk of bias due to missing results in a synthesis (arising from reporting biases). | Page 6 |
| Certainty assessment | 15 | Describe any methods used to assess certainty (or confidence) in the body of evidence for an outcome. | Page 5 |
| **RESULTS** | | |  |
| Study selection | 16a | Describe the results of the search and selection process, from the number of records identified in the search to the number of studies included in the review, ideally using a flow diagram. | Page 6,7 |
|  | 16b | Cite studies that might appear to meet the inclusion criteria, but which were excluded, and explain why they were excluded. | Page 6,7 |
| Study characteristics | 17 | Cite each included study and present its characteristics. | Page 7-9 |
| Risk of bias in studies | 18 | Present assessments of risk of bias for each included study. | Page 12-14 |
| Results of individual studies | 19 | For all outcomes, present, for each study: (a) summary statistics for each group (where appropriate) and (b) an effect estimate and its precision (e.g. confidence/credible interval), ideally using structured tables or plots. | Page 7-9 |
| Results of syntheses | 20a | For each synthesis, briefly summarise the characteristics and risk of bias among contributing studies. | Page 9-11 |
|  | 20b | Present results of all statistical syntheses conducted. If meta-analysis was done, present for each the summary estimate and its precision (e.g. confidence/credible interval) and measures of statistical heterogeneity. If comparing groups, describe the direction of the effect. | Page 9-11 |
|  | 20c | Present results of all investigations of possible causes of heterogeneity among study results. | Page 11 |
|  | 20d | Present results of all sensitivity analyses conducted to assess the robustness of the synthesized results. | Page 12 |
| Reporting biases | 21 | Present assessments of risk of bias due to missing results (arising from reporting biases) for each synthesis assessed. | - |
| Certainty of evidence | 22 | Present assessments of certainty (or confidence) in the body of evidence for each outcome assessed. | Page 14-16 |
| **DISCUSSION** | | |  |
| Discussion | 23a | Provide a general interpretation of the results in the context of other evidence. | Page 18-20 |
|  | 23b | Discuss any limitations of the evidence included in the review. | Page 20 |
|  | 23c | Discuss any limitations of the review processes used. | Page 20 |
|  | 23d | Discuss implications of the results for practice, policy, and future research. | Page 19 |
| **OTHER INFORMATION** | | |  |
| Registration and protocol | 24a | Provide registration information for the review, including register name and registration number, or state that the review was not registered. | Page 2 |
|  | 24b | Indicate where the review protocol can be accessed, or state that a protocol was not prepared. | A protocol was not prepared |
|  | 24c | Describe and explain any amendments to information provided at registration or in the protocol. | - |
| Support | 25 | Describe sources of financial or non-financial support for the review, and the role of the funders or sponsors in the review. | Page 29 |
| Competing interests | 26 | Declare any competing interests of review authors. | Page 28 |
| Availability of data, code and other materials | 27 | Report which of the following are publicly available and where they can be found: template data collection forms; data extracted from included studies; data used for all analyses; analytic code; any other materials used in the review. | Page 28 |

*From:*  Page MJ, McKenzie JE, Bossuyt PM, Boutron I, Hoffmann TC, Mulrow CD, et al. The PRISMA 2020 statement: an updated guideline for reporting systematic reviews. BMJ 2021;372:n71. doi: 10.1136/bmj.n71

**Search strategy:**

Pubmed

#1：((((((((((((((((((Emergence Delirium[MeSH Terms]) OR (Delirium, Emergence[Title/Abstract])) OR (Emergence Agitation[Title/Abstract])) OR (Agitation, Emergence[Title/Abstract])) OR (Agitations, Emergence[Title/Abstract])) OR (Post-Operative Delirium[Title/Abstract])) OR (Delirium, Post-Operative[Title/Abstract])) OR (Post Operative Delirium[Title/Abstract])) OR (Postanesthetic Excitement[Title/Abstract])) OR (Excitement, Postanesthetic[Title/Abstract])) OR (Anesthesia Emergence Delirium[Title/Abstract])) OR (Delirium, Anesthesia Emergence[Title/Abstract])) OR (Emergence Delirium, Anesthesia[Title/Abstract])) OR (Postoperative Delirium[Title/Abstract])) OR (Delirium, Postoperative[Title/Abstract])) OR (Agitated Emergence[Title/Abstract])) OR (Emergence, Agitated[Title/Abstract])) OR (Emergence Excitement[Title/Abstract])) OR (Excitement, Emergence[Title/Abstract])

#2：((((Hypotension[MeSH Terms]) OR (Vascular Hypotension[Title/Abstract])) OR (Low Blood Pressure[Title/Abstract])) OR (Blood Pressure, Low[Title/Abstract])) OR (Hypotension, Vascular[Title/Abstract])

#3：#1 and #2

Embase

('hypotension'/exp OR 'acute hypotension':ti,ab OR 'arterial hypotension':ti,ab OR 'blood pressure, low':ti,ab OR 'hypotension':ti,ab OR 'hypotension syndrome':ti,ab OR 'hypotensive syndrome':ti,ab OR 'low blood pressure':ti,ab) AND ('postoperative delirium'/exp OR 'delirium after surgery':ti,ab OR 'delirium after surgical intervention':ti,ab OR 'delirium after surgical repair':ti,ab OR 'delirium after surgical resection':ti,ab OR 'post-operative confusion':ti,ab OR 'post-operative deliria':ti,ab OR 'post-operative delirium':ti,ab OR 'postoperative confusion':ti,ab OR 'postoperative deliria':ti,ab OR 'postoperative delirious state':ti,ab OR 'postoperative delirium':ti,ab)

Cochran

#1：MeSH descriptor: [Hypotension] explode all trees

#2：(Blood Pressure, Low or Low Blood Pressure or Vascular Hypotension or Hypotension, Vascular):ti,ab,kw

#3：MeSH descriptor: [Emergence Delirium] explode all trees

#4：(Excitement, Postanesthetic or Post-Operative Delirium or Excitement, Emergence or Delirium, Post-Operative or Anesthesia Emergence Delirium or Emergence Delirium, Anesthesia or Emergence Agitation or Post Operative Delirium or Delirium, Anesthesia Emergence or Delirium, Emergence or Emergence, Agitated or Postanesthetic Excitement or Emergence Excitement or Delirium, Postoperative or Agitations, Emergence or Postoperative Delirium or Agitation, Emergence or Agitated Emergence):ti,ab,kw

#5：#1 or #2

#6：#3 or #4

#7：#5 and #6

Wos

(TS=(hypotension) OR AB=(Vascular Hypotension OR Low Blood Pressure OR Blood Pressure, Low OR Hypotension, Vascular)) AND (TS=(Emergence Delirium) OR AB=(Delirium, Emergence OR Emergence Agitation OR Agitation, Emergence OR Agitations, Emergence OR Post-Operative Delirium OR Delirium, Post-Operative OR Post Operative Delirium OR Postanesthetic Excitement OR Excitement, Postanesthetic OR Anesthesia Emergence Delirium OR Delirium, Anesthesia Emergence OR Emergence Delirium, Anesthesia OR Postoperative Delirium OR Delirium, Postoperative OR Agitated Emergence OR Emergence, Agitated OR Emergence Excitement OR Excitement, Emergence))

Table S1. Univariate Meta-regression for Intraoperative Hypotension and Postoperative Delirium: Moderator Effects on Heterogeneity (Tau & I²)

| Moderator | Tau with Moderator | I² with Moderator (%) | Tau without Moderator | I² without Moderator (%) | Change in Tau | Change in I² |
| --- | --- | --- | --- | --- | --- | --- |
| None (No Moderator) | 0.9526 | 94.14 | 0.9526 | 94.14 | Baseline | Baseline |
| Study type （cohort vs case-control） | 0.9438 | 92.28 | 0.9526 | 94.14 | Decrese | Decrese |
| Threshold Nature | 0.8069 | 88.94 | 0.9526 | 94.14 | Decrese | Decrese |
| Blood Pressure Parameter | 1.0677 | 95.09 | 0.9526 | 94.14 | Increase | Increase |
| Screening tool of delirium | 1.0397 | 92.28 | 0.9526 | 94.14 | Increase | Decrese |
| Region | 0.8823 | 91.81 | 0.9526 | 94.14 | Decrese | Decrese |

Table S2. Univariate Meta-regression for Intraoperative Hypotension and Postoperative Delirium: Contrast Table Including the Overall Estimate With Versus Without Moderators

| Moderator | Adjusted OR (95% CI)^a^ | p-value | Overall Estimate Without Moderators (OR, 95% CI)^b^ | Change^c^ |
| --- | --- | --- | --- | --- |
| None | 2.54 (1.45 - 4.45) | 0.001 | 2.54 (1.45 - 4.45) | Baseline |
| Study type （cohort vs case-control） | 2.59 (1.20 – 5.58) | 0.273 | 2.54 (1.45 - 4.45) | Minimal Increase |
| Threshold Nature | 2.86 (1.41 – 5.82) | 0.048 | 2.54 (1.45 - 4.45) | Increase |
| Blood Pressure Parameter | 2.81 (1.09 – 7.22) | 0.852 | 2.54 (1.45 - 4.45) | Increase |
| Screening tool of delirium | 2.56 (0.94 – 6.96) | 0.812 | 2.54 (1.45 - 4.45) | Minimal Increase |
| Region | 2.67 (1.11 – 6.46) | 0.177 | 2.54 (1.45 - 4.45) | Minimal Increase |

a: Reflects the odds ratio after including the moderator in the meta-regression model.

b: The baseline odds ratio and its significance before including any moderator.

c: Describes whether the inclusion of the moderator increased or decreased the estimate compared to the baseline.
